# Supplementary material for: Bioactive Efficacy of Novel Carboxylic Acid from Halophilic Pseudomonas aeruginosa against Methicillin-Resistant Staphylococcus aureus
Source: Metabolites. 2022 Nov 10;12(11):1094. doi: 10.3390/metabo12111094 (PMC9698732; doi:10.3390/metabo12111094)
Supplement: Supplementary file 1 [file metabolites-12-01094-s001.zip › metabolites-1997536-supplementary.pdf]

Supplementary Materials:

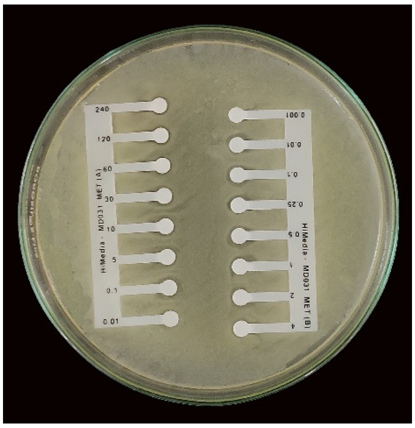

Figure S1. Methicillin resistance in hospital-derived *S. aureus* (MRSA) in MHA plate using methicillin antibiotic strip.

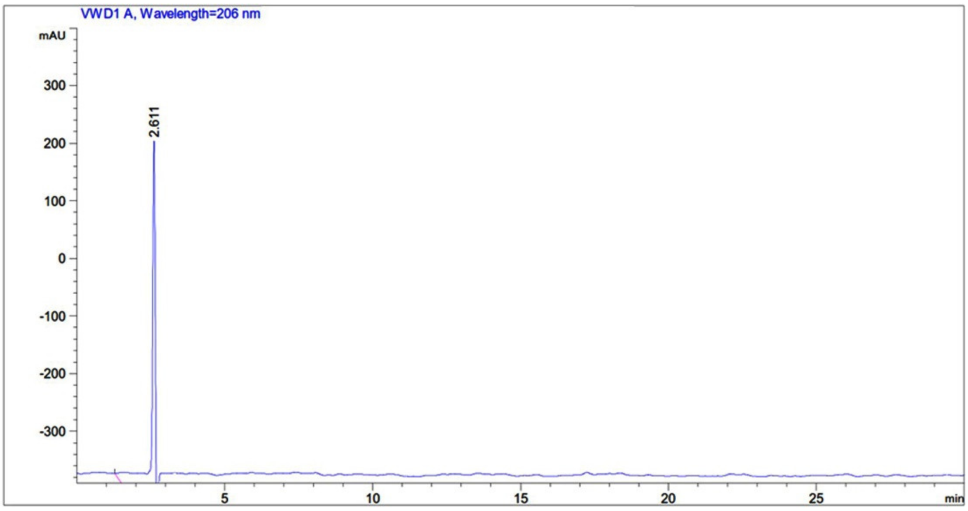

Figure S2. HPLC data of isolated compound 5-(1*H*-indol-3-yl)-4-pentyl-1,3-oxazole-2-carboxylic acid (Compound **1**).

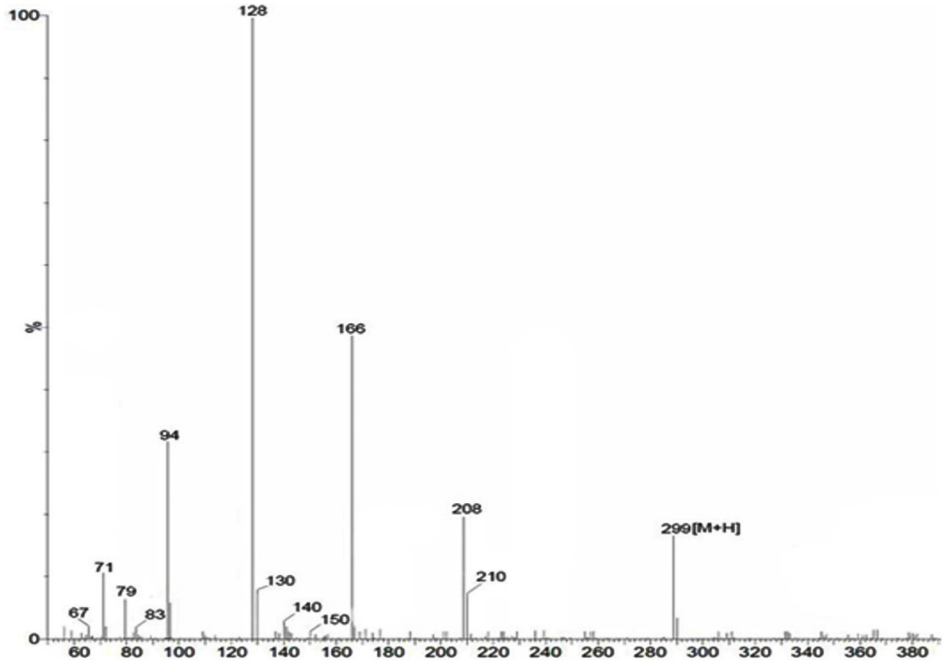

Figure S3. LC/MS spectrum of 5-(1*H*-indol-3-yl)-4-pentyl-1,3-oxazole-2-carboxylic acid (Compound **1**).

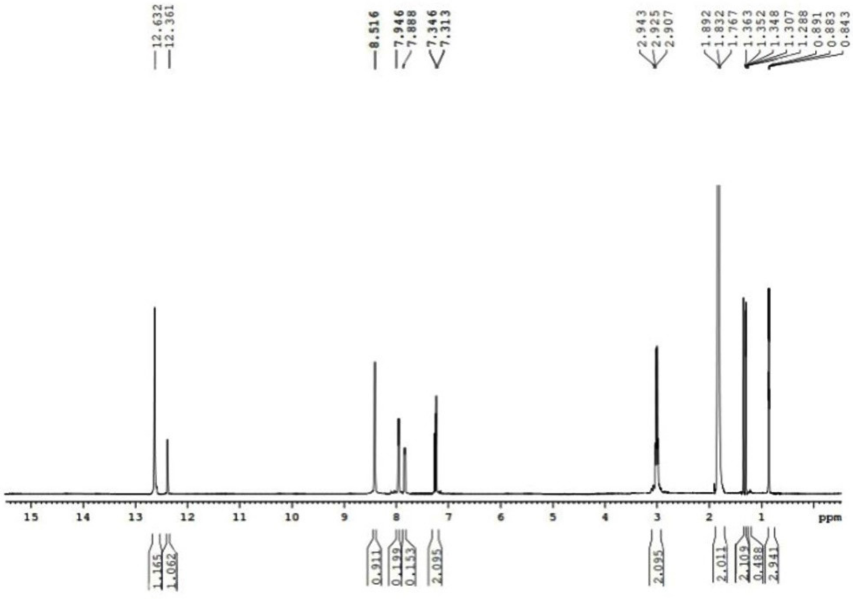

Figure S4a. ^{1}H-NMR spectrum of 5-(1*H*-indol-3-yl)-4-pentyl-1,3-oxazole-2-carboxylic acid (Compound **1**).

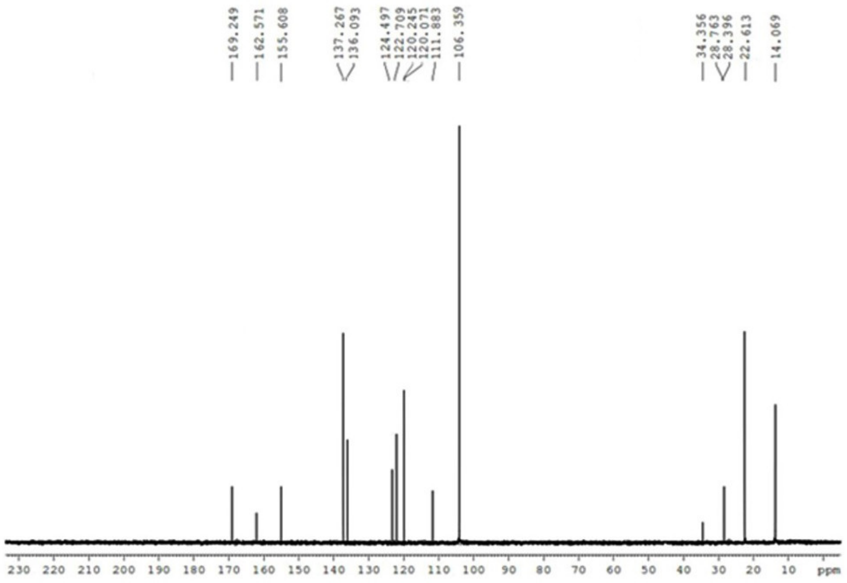

Figure S4b. ^{13}C-NMR spectrum of 5-(1*H*-indol-3-yl)-4-pentyl-1,3-oxazole-2-carboxylic acid (Compound **1**).

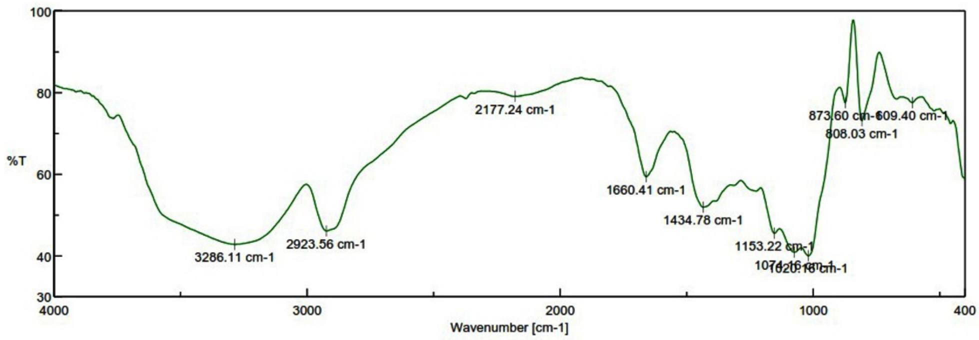

Figure S5. IR spectrum of 5-(1*H*-indol-3-yl)-4-pentyl-1,3-oxazole-2-carboxylic acid (Compound **1**).
